# Impact of dedicated hemostasis device for distal radial arterial access with an adequate hemostasis protocol on radial arterial observation by ultrasound

**DOI:** 10.1007/s12928-020-00656-4

**Published:** 2020-03-12

**Authors:** Yota Kawamura, Fuminobu Yoshimachi, Norihito Nakamura, Yoshiya Yamamoto, Takeaki Kudo, Yuji Ikari

**Affiliations:** 1 Division of Cardiology, Department of Internal Medicine, Tokai University Hachioji Hospital, 1838 Ishikawa-cho Hachioji, Tokyo, 192-0032 Japan; 2 Division of Cardiology, Department of Internal Medicine, Tokai University School of Medicine, Isehara, Japan

**Keywords:** Distal radial artery (dRA), Hemostasis, Radial artery occlusion (RAO)

## Abstract

There is no established hemostasis method or protocol for the transdistal radial approach. Therefore, this study aimed to determine whether “the PreludeSYNC DISTAL” radial compression device (PSD; Merit Medical Systems, Inc., South Jordan, UT) can effectively prevent distal radial artery (dRA) occlusion following catheterization procedures. This retrospective study analyzed patients who underwent hemostasis using the PSD from January 1, 2019, to March 31, 2019. The primary endpoint was occlusion or excessive stenosis of the radial artery (RA) 1 month after catheterization. Pulsatile blood flow and vessel diameters of the dRA and forearm RA (fRA) were measured using vascular ultrasound before and 1 month after catheterization to determine arterial damage. Secondary endpoints were achievement of hemostasis, bleeding, hematoma, aneurysm, neurological abnormality, and functional disturbance of the fingers or hand. Fifty patients (mean age, 70.9 ± 10.7 years; male, 72.0%) were enrolled in this study. Complete hemostasis was achieved in all cases. Total hemostasis time was 161 ± 45 min. No procedure-associated complications were noted. Pulsations of the dRA and fRA were maintained at 1 month. No functional disturbance or neurological abnormality was observed. Vessel diameters of the dRA and fRA were not significantly different before and 1 month after catheterization. No dissection, pseudoaneurysm, or occlusion/stenosis was observed on ultrasound. Distal radial access with a unique device and protocol effectively achieved hemostasis and prevented injury and occlusion of the dRA and fRA.

## Introduction

Percutaneous coronary intervention (PCI) is an established treatment for ischemic heart disease [1–3]. A factor that affects the prognosis of PCI is the site used during the approach. The transfemoral approach (TFA) was the main approach; however, studies have indicated that the transradial approach (TRA) is more advantageous because of its fewer bleeding complications, lesser discomfort with joint mobility, lesser restriction of behavior, and better prognosis than TFA [4, 5]. However, radial artery (RA) occlusion (RAO) is a serious problem of TRA even in the absence of clinical symptoms. Several factors including long hemostasis time and the ratio of the RA diameter and sheath have been reported to cause RAO [6]. Therefore, sheaths with a smaller diameter and an adequate hemostasis device that can shorten the hemostasis time are necessary to effectively prevent RAO [7, 8].

Recently, a new approach involving coronary catheterization via the distal RA (dRA) at the anatomical snuffbox has been reported [9]. An advantage of this method is its shorter hemostasis time than conventional TRA [10, 11]. However, no established hemostasis method or protocol for the transdistal radial approach and no suitable method of hemostasis using dRA access and a dedicated device have been reported.

**Fig. 1:**
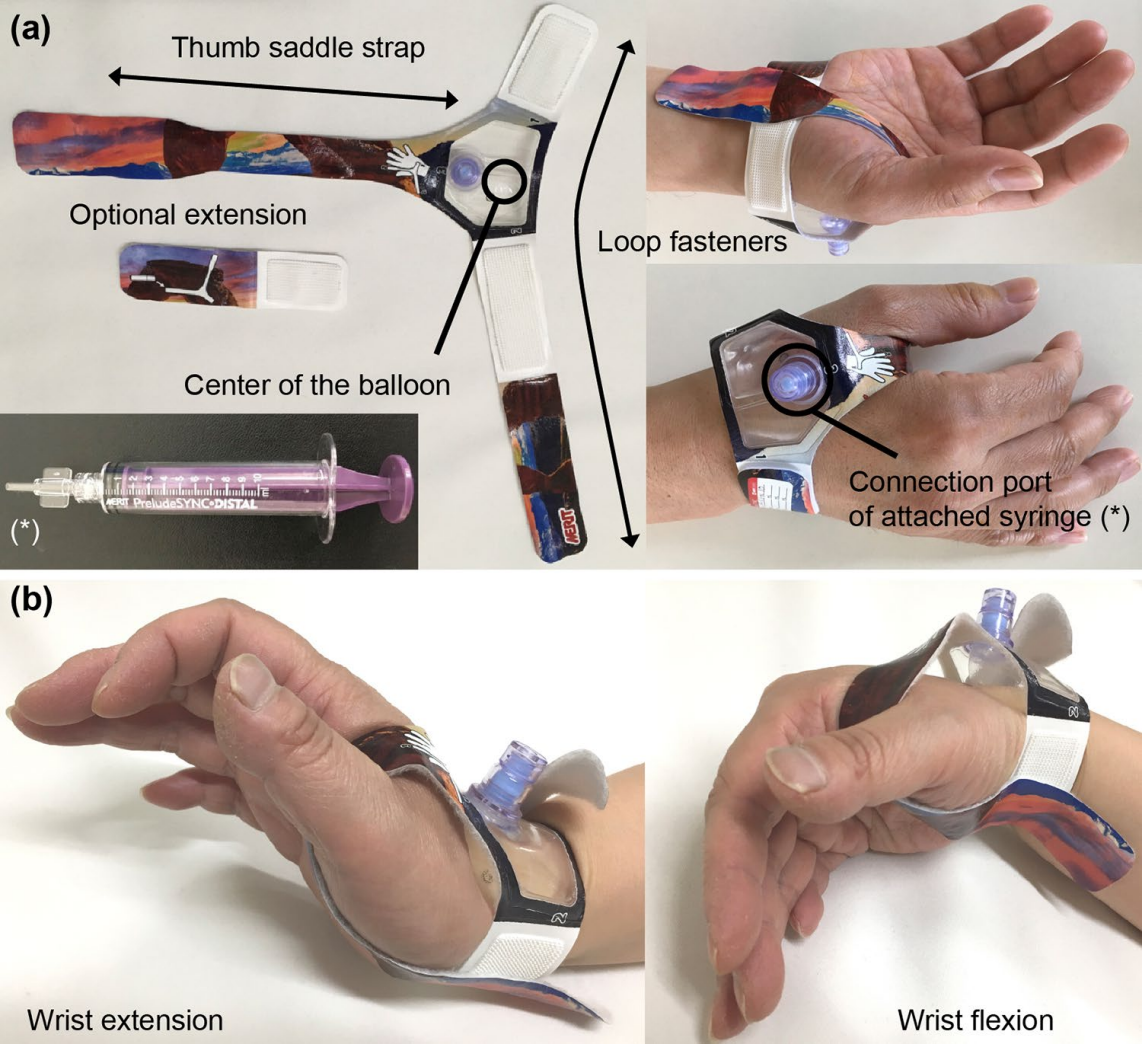
Appearance of the PreludeSYNC DISTAL radial compression device. **a** The appearance of new compression device for distal radial artery: the PreludeSYNC DISTAL radial compression device (Merit Medical Systems, Inc. South Jordan, UT) is composed of a soft wristband, thumb saddle strap with loop fasteners, and inflatable balloon with an attached syringe to compress the puncture site. **b** Hemostasis device fixation under wrist bending. The PreludeSYNC DISTAL radial compression device is not practically misaligned despite wrist bending

A new compression hemostasis device, the PreludeSYNC DISTAL radial compression device (PSD; Merit Medical Systems, Inc., South Jordan, UT) was developed exclusively for the dRA approach (Fig. 1a). This device became available in Japan in February 2019. It is a disposable, dedicated hemostasis device that compresses the punctured dRA site with an inflatable balloon. However, the effectiveness of this device and its proper use has not been reported. To prevent injury and occlusion of the dRA and forearm RA (fRA), a new protocol was created at our hospital and performed during hemostasis of the dRA.

During this study, the effectiveness of the PSD and our protocol for preventing dRA or fRA injury and occlusion was researched retrospectively.

## Methods

### Case selection

**Fig. 2:**
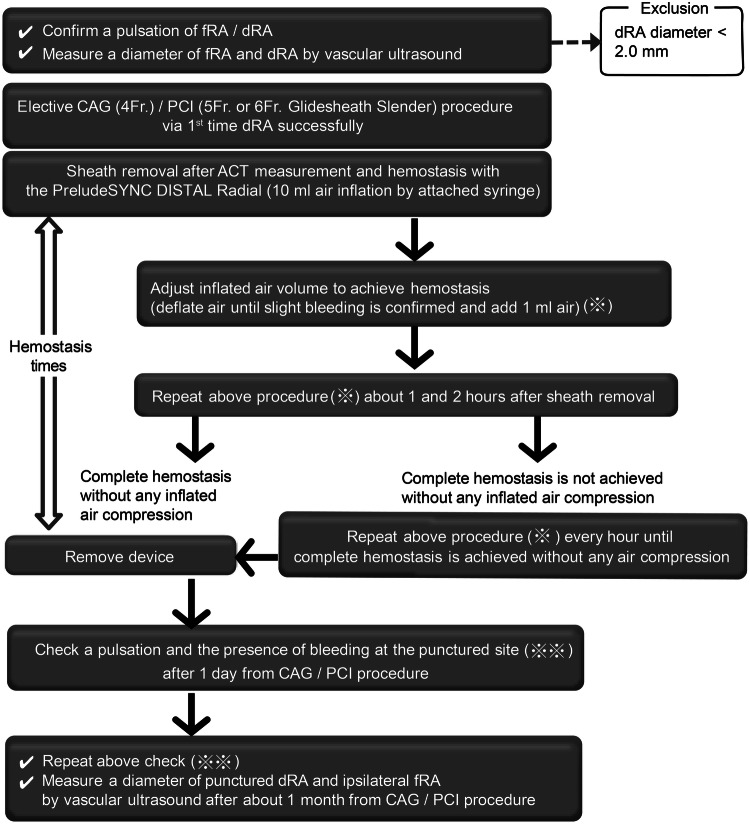
Hemostasis protocol with the PreludeSYNC DISTAL radial compression device and observational procedure

This was a sequential retrospective observational study performed at a single center. Patients who underwent coronary catheterization via the dRA for the first time and with the use of the PSD as a hemostasis device from January 1, 2019, to March 31, 2019 were enrolled (Fig. 2). Patients underwent diagnostic coronary angiography (CAG) or PCI according to the usual indications. Moreover, patients underwent cannulation via the radial or distal radial approach, except those on dialysis or requiring dialysis in the near future. Arterial diameters of the fRA and dRA before the coronary catheterization procedure were measured using vascular ultrasound. Cross-sectional diameters of the fRA and dRA at the point of puncture were measured, and the dRA approach with a 5 Fr sheath of a small diameter or a 4 Fr conventional sheath was selected only for patients with dRA diameters of > 2.0 mm to avoid injury from the large sheath. If a 6 Fr sheath is needed because of the strategy, then the diameter of the artery would needed to be > 2.5 mm. Exclusion criteria were acute coronary syndrome requiring emergent PCI, failed dRA puncture, sheath insertion from the dRA, and difficulty scheduling follow-up visits.

All patients provided informed written consent, and the study conformed to institutional ethics guidelines and those of the American Physiological Society.

### Coronary catheterization procedure

After the administration of local anesthesia, a disposable plastic-cannulated needle was inserted in the dRA with pulse palpable or vascular ultrasound using the Seldinger technique, and a hydrophilic-coated wire attached to the sheath system was inserted carefully. A sheath with an adequate size for the procedure was inserted. A 30 cm 4 Fr sheath (Supersheath, Medikit, Tokyo, Japan) was used for CAG and a 16 cm 5 Fr or 6 Fr sheath [Glide Sheath Slender (GSS); Terumo, Tokyo, Japan] [12] for PCI. Unfractionated heparin was administered intra-arterially via the sheath. The initial injected dose of unfractionated heparin was 2000 units for CAG and 5000 units for PCI. Additional 2000 units of unfractionated heparin were administered every hour. After finishing the procedures, the activated clotting time (ACT) was measured before sheath removal. These procedures were performed by various physicians with experience performing coronary catheterization procedures via the dRA.

### Hemostasis of the dRA after CAG or PCI using the PSD

The PSD for hemostasis was used for dRA hemostasis after coronary catheterization as follows:

1. Clean and dry the dRA site with ethanol.
2. Prepare the PSD for the right hand or left hand as appropriate.
3. Withdraw the sheath at approximately 1 inch.
4. Place the PSD using the black spot as the landmark at the center of the balloon to access the puncture site.
5. Tightly fasten the band around the wrist.
6. Wrap band between the thumb and forefinger without slack.
7. Fill the attached exclusive syringe with 10 mL of air and connect the valve on top of the device.
8. Slowly inflate the balloon with air while simultaneously removing the sheath. When the sheath is completely removed, continue injecting air in the balloon until hemostasis is complete.
9. Adjust the inflated air volume to achieve hemostasis (deflate air until slight bleeding is confirmed and add 1 mL of air using the syringe).
10. Disconnect the syringe.
11. Approximately 1 h after sheath removal, adjust the inflated air volume to achieve hemostasis as in step 9.
12. Approximately 2 h after sheath removal, adjust the inflated air volume to achieve hemostasis as in step 9.
13. Remove the PSD if complete hemostasis is achieved without inflated air compression. If complete hemostasis is not achieved, then repeat step 9 every hour until complete hemostasis is achieved.
14. Record the time and inflated air volume of each process.
15. During the hemostasis period, there is no restriction of wrist movement (Fig. 1b).

### Follow-up of the punctured dRA site

Pulsation and the presence of complications associated with hemostasis procedure (detailed below) were checked after 1 day and approximately 1 month after the procedure. At 1 month follow-up examination, patency of the artery and arterial diameters of the fRA and dRA were measured by vascular ultrasound using the same method prior to coronary catheterization.

### Endpoints

The primary endpoint was occlusion or excessive stenosis of the RA at 1 month after catheterization. Pulsatile blood flow and the vessel diameters of the dRA and fRA using vascular ultrasound were examined. Arterial diameters of the fRA and dRA were compared with those before coronary catheterization using Student’s *t* test and SPSS software version 25 (IBM Corp., Armonk, NY).

Secondary endpoints were as follows: successful and safe hemostasis at the dRA puncture site; hemostasis time; vascular complications associated with the procedure, including bleeding, hematoma and pseudoaneurysm; neurological abnormality; and functional disturbances of the fingers or hand.

Hemostasis time was defined as the period from sheath removal to complete detachment of the PSD. Bleeding events were defined according to the BARC criteria [13]. A minor hematoma was defined as < 3.0 cm without symptoms, and a major hematoma was defined as > 3.0 cm or the presence of symptoms caused by the hematoma. The existence of a pseudoaneurysm was checked by ultrasound at approximately 1 month after the procedure. Neurological sequelae were defined as symptomatic abnormal feelings associated with the hemostasis procedure. Functional disturbance was defined as paralysis or disorders of hand and/or finger movements caused by the hemostasis procedure.

## Results

Fifty patients were enrolled consecutively during this study. Background characteristics and coronary catheterization procedures are shown in Table 1. The mean age was 70.9 ± 10.7 years, and 72.0% patients were men. Thirty two patients underwent CAG (4 Fr sheath for 31 patients; 5 Fr sheath for 1 patient). PCI was performed for 18 patients (15 with the 5 Fr GSS and 3 with the 6 Fr GSS). Each dRA diameter of patients who underwent PCI with the 6 Fr GSS was > 3.0 mm. Twenty-one patients received dual antiplatelet therapy (DAPT). Nine patients received anticoagulant therapy, including five patients who received both DAPT and anticoagulant therapy. The mean adjusted inflated air volume after sheath removal at the start of hemostasis was 8.2 ± 1.3 mL.

**Table 1:** Background of patients and coronary catheterization procedures

Number of cases	50
Mean age (years)	70.9 ± 10.7
Male sex (%)	36 (72.0%)
Body mass index (kg/m^2^)	24.1 ± 4.1
Prevalence of coronary risk factor	
Hypertension	35 (70.0%)
Dyslipidemia	39 (78.0%)
Diabetes	22 (44.0%)
Current smoker	23 (46.0%)
Chronic kidney disease	4 (8.0%)
Antiplatelet therapy	
Aspirin	38 (76.0%)
Clopidogrel	22 (44.0%)
Prasugrel	9 (18.0%)
Dual antiplatelet therapy	21 (42.0%)
Anticoagulant therapy	
Warfarin	3 (6.0%)
Direct oral anticoagulants	6 (12.0%)
Dual antiplatelet plus anticoagulant therapy	5 (10.0%)
Coronary catheterization	
Coronary angiography	32 (64.0%)
Coronary intervention	18 (36.0%)
Sheath size (%)	
4-F	31 (62.0%)
5-F GSS	16 (32.0%)
6-F GSS	3 (6.0%)
Heparin administration (U)	3340.0 ± 1901.8
Activated clotting time at sheath removal (s.)	252.9 ± 54.1
Inflated air volume after sheath removal (mL)	8.2 ± 1.3

*GSS* glidesheath slender

The mean duration of hemostasis time was 161 ± 45 min (Table 2). There were no complications associated with the hemostasis procedure. No occlusions of the dRA were observed after removing the PSD or 1 day after the procedure, which was the day of discharge.

**Table 2:** Hemostasis and complications on the day after catheterization

Duration of hemostasis (min)	161 ± 45
dRA pulsation (%)	50 (100%)
fRA pulsation (%)	50 (100%)
Complication related to hemostasis procedure (%)	
Bleeding (any type of BARC definition)	0 (0%)
Major hematoma	0 (0%)
Minor hematoma	0 (0%)
Pseudoaneurysm	0 (0%)
Neurological sequela	0 (0%)

*BARC* Bleeding Academic Research Consortium, *dRA* distal radial artery, *fRA* forearm radial artery

**Fig. 3:**
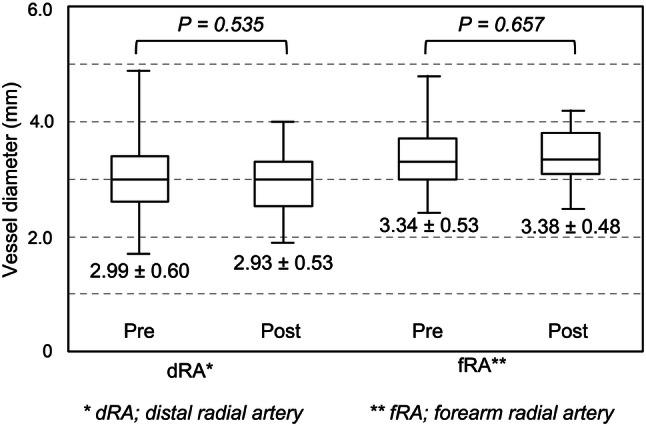
Diameter of the distal radial artery and forearm radial artery compared before and after catheterization

No occlusions, stenoses, pseudoaneurysms, or dissections of the dRA and fRA were observed on ultrasound approximately 1 month after catheterization. No functional disturbance was observed. Vessel diameters of the dRA and fRA before and after catheterization were compared, and no significant differences were observed between these measurements (Fig. 3).

## Discussion

This is the first report of the dRA condition observed using ultrasound and a patent hemostasis protocol with a dedicated hemostasis device after the transdistal radial approach to coronary catheterization. Using the PSD and a simple hemostatic protocol, blood flow in both the dRA and fRA without damage, as observed using ultrasound was maintained 1 month after catheterization and complete hemostasis without complications was achieved in all cases.

Conventional TRA has been the standard method because of its many advantages including lower rates of local vascular complications such as hematoma and pseudoaneurysm, less bleeding, better comfort, lower costs, shorter hospital stays, and decreased workload for nursing staff [14]. Nevertheless, TRA also has some serious and unresolved problems, including the limitation of the sheath size and RAO, which limits the fRA as a future access site and disturbs creating the shunt for hemodialysis. The reported occlusion rate of the fRA after TRA is 4–12% [15–17]. The main causes are fRAs with a small diameter and the hemostasis method.

However, the transdistal radial approach for coronary catheterization has a relatively shorter history than the TRA. Therefore, many issues associated with the transdistal radial approach have not yet been resolved. Generally, the dRA is smaller than the fRA and has many anatomical variations because of its peripheral location. Transradial interventions became safer after studying hemostatic devices and their proper use. Therefore, to further pursue the benefits of using the dRA and to avoid complications of dRA injuries and occlusions, a slender catheter and an adequate hemostasis method with a dedicated hemostasis device are needed to improve the distal radial approach.

The long history of the conventional radial approach has given insights into how to avoid damage to the artery, it is necessary to use a sheath smaller than the vessel diameter to achieve a secure puncture and gradually decompress and release the hemostasis device early to avoid RAO.

Before the launch of the PSD as the first dedicated hemostasis device for the distal radial approach, various hemostasis methods involving elastic tape and bandages have been suggested. These techniques had a learning curve and it was impossible to calculate the fixing power of the bandages for each patient. Furthermore, their uniqueness has made it difficult to establish standard hemostasis protocols. In contrast, the PSD is simple to use with a uniform protocol for dRA hemostasis.

During this study, no bleeding complications were observed, and blood flow in the dRA was maintained at 1 month after the procedure in all cases. These results demonstrate that our hemostatic protocol with pressure reduction of the PSD to the greatest extent possible and more than once effectively avoided arterial occlusion.

Excluding cases with dRA of diameters < 2.0 mm may have affected the results of this study. The sensitivity and specificity for detecting severe flow reduction of the fRA versus the ratio of the fRA diameter to the sheath outer diameter were between 1.0 and 1.1 in the previous study, respectively [6, 18]. Therefore, a threshold of 2.0 mm was determined to be the threshold diameter for the distal radial approach using the 5 Fr GSS [12]. Of course, if the dRA and fRA diameters are larger than the outer diameter of the sheath, then a sheath with a larger diameter could be inserted. However, using a sheath with a larger diameter may increase the hemostasis time, as has been experienced with conventional transradial or transfemoral intervention, and this may cause bleeding complications or occlusion of the artery. Therefore, adequate case selection in accordance with our protocol and the selection of an appropriate sheath size may have contributed to the good outcomes obtained in our study.

The ultrasound, which is a non-invasive examination modality, was used at all catheterization sites to measure the vessel diameter and to observe the condition of the artery. We believe that cannulation of a vessel without measuring the size or recognizing the location of the vessel should not be performed. Ultrasound-guided puncture is recommended and considered an excellent step when using the conventional radial, femoral, and distal radial approaches [19–22]. The fRA is an access site in hemodialysis patients that should be avoided because RAO, which is a serious problem, can occur when creating vascular access. However, the transdistal radial approach for coronary catheterization and our hemostasis protocol may be useful for hemodialysis and avoid fRA and dRA occlusion. Because the dRA occlusion ratio may be low and fRA is located proximal site of the bifurcation into the dRA and ramus volaris superficialis, fRA may not be occluded.

The PSD hemostasis device has many features. The transparent balloon may contribute to the early detection of recurrent bleeding by nurses or patients. Its soft wristband and thumb saddle strap also contribute to secure hemostasis. The puncture site of the dRA is located in the snuffbox near the carpometacarpal joint of the thumb. Previously, with classical bandage hemostasis after sheath removal, movement of the carpometacarpal joint caused misalignment of the puncture point and hemostasis device, possibly preventing stable pressurization. Furthermore, movement of the carpometacarpal joint, including palmar abduction and adduction, radial abduction, and ulnar adduction, might also cause misalignment of the bandage. Compared with other balloon inflation type devices, the PSD has the advantage of being dedicated to the dRA. Its most remarkable characteristic is the thumb saddle strap that prevents longitudinal misalignment that can occur with various movements of the carpometacarpal joint.

No patients in this study had recurrent bleeding from the puncture site during hemostasis with the PSD despite unrestricted wrist movement during the hemostasis period (Fig. 1b). This suggests that hemostasis of the punctured dRA site using the PSD is more comfortable for patients than any classical method for the dRA or other conventional TRA that dose not allow patients to move the wrist during hemostasis.

One of the disadvantages of the PSD is its cost. Hemostasis with a simple bandage is less expensive than using a new exclusive device. The cost-effectiveness of each device must be considered in the context of the increasing costs of healthcare.

### Study limitations

This study had several limitations. First, it was a single-center retrospective study that included a small number of patients. Second, the hemostasis protocol and hemostasis device were not compared with others. Third, the specific sheath had a limited size of < 6 Fr, and was used during daily angiography and PCI. A multicenter, prospective registry including the use of larger sheaths for coronary catheterization and comparing this hemostasis protocol with other protocols and hemostasis devices is expected to demonstrate the superiority of our hemostasis protocol. Fourth, patients with dRA diameters of the dRA < 2.0 mm were excluded. If this threshold was changed, then different results might be obtained. Using this threshold, the distal radial approach was performed for approximately 50% of the total transradial interventions during our daily practice. This must be revised to achieve the benefits of dRA in more patients. Fifth, the arterial diameter was measured during vascular ultrasound examinations by several physicians in this study. Therefore, measurement mismatches were possible to due to subjectivity.

## Conclusion

An adequate hemostatic protocol with the PSD led to blood flow maintenance in both the dRA and fRA without damage at 1 month after catheterization. Complete hemostasis without complications was achieved in all cases. If the issue of cost is solved, then patent hemostasis methods using the PSD could become the standard procedure for puncturing dRA sites.
